# Targeting FANCD2 for therapy sensitization

**DOI:** 10.18632/oncotarget.2070

**Published:** 2014-06-07

**Authors:** Changxian Shen, Peter J. Houghton

**Affiliations:** Center for Childhood Cancer and Blood Diseases, Nationwide Children's Hospital, Columbus, OH

The Fanconi Anemia (FA) signaling pathway is essential for the maintenance of genome integrity and cells to survive DNA interstrand crosslink (ICL) by coordinating DNA damage repair through translesion DNA synthesis (TLS), nucleotide excision repair (NER) and homologous recombination (HR). Besides ICL, the FA signaling pathway is activated by different kinds of genotoxins and plays an important role in the activation of the ATM DNA damage and ATR intra-S phase checkpoints. There are fifteen FANC genes identified in FA or FA-like patients. FA-pathway deficient cells display spontaneous DNA strand breaks under normal growth conditions and defect of DNA damage checkpoint activation in response to DNA damage or replication stress [1]. FANCD2 is the critical component of FA signaling. In response to ICL, the FA pathway activates the FA core E3 ubiquitin ligase complex, which in turn leads to monoubiquitination of FANCI and FANCD2. Monoubiquitinated FANCI-FANCD2 complex is recruited to DNA damage sites and helps endonucleases to cut both sides of ICL to generate DNA strand breaks, and promotes TLS, NER and Rad51-medated HR [2, 3]. The molecular mechanisms by which FA signaling maintains genome stability, coordinates multiple DNA damage repair pathways and facilitates the activity of ATM/ATR checkpoints, remain to be determined. We have recently reported [4] that FANCD2 is required for the timely ATM-Chk2 activation in the early steps of FA signaling-mediated repair of ICL-induced DNA lesions [5]. In rhabdomyosarcoma Rh30 cells, we found that during the early response to ICL FANCD2 is required for the proper phosphorylation of H2AX and hence activation of ATM, but not essential for ATR-Chk1 activation, supporting the proposed model of the function of FANCD2 in response to ICL [2, 3]. The ATM DNA damage checkpoint maintains the integrity of genetic information under normal growth and cell survival in response to DNA double strand breaks [6]. Our findings suggest that FANCD2 dependent activation of the ATM checkpoint in the early response to ICL is one of the mechanisms by which FA signaling promotes genome stability under normal growth condition and cell survival in response to genotoxins.

Most cancers have deregulated the PI3K and Ras signaling pathways, both of which converge on the mammalian target of rapamycin (mTOR). Molecularly targeting PI3K or Ras signaling sensitizes many cancers to radiotherapy and chemotherapy [7]. Moreover, in yeast, TOR promotes cell survival but at the cost of increased mutation rate in response to DNA damage agents [8]. We found that an mTOR kinase inhibitor sensitizes mouse rhabdomyosarcoma Rh30 tumor xenografts to ionizing radiation and cultured Rh30 cells to the bifunctional alkylating agent melphalan, accompanied with significant downregulation of FANCD2. Further pharmacological and genetic analysis demonstrated that mTOR signaling controls the gene transcription of *FANCD2* via CDK4, supporting the observation that *FANCD2* is regulated by Rb-E2F1 [9]. During the multiple step processes of tumorigenesis, most cancer cells have acquired defects in the cell cycle checkpoint to increase the activity of CDK4/6 by disrupting PI3K, Ras, p53 and Rb signaling circuits. Positive control of FANCD2 by CDK4 suggests that cancer cells with self-sufficiency in growth signaling and resistance to anti-proliferation signaling may depend on FANCD2 for survival.

Chemotherapeutic agents that damage DNA and radiotherapy are the major components of current clinical protocols for the treatment of childhood cancers. Among them, DNA interstrand crosslink agents such as cisplatin, cyclophosphamide, and melphalan, are the first line drugs for the treatment of pediatric cancers, but many patients develop resistance to these drugs later. Regarding the essential roles of FANCD2 in the resistance of cells to ICL and activation of DNA damage checkpoints, our findings provide a strategy to cancer therapy by targeting FANCD2 through molecular inhibition of PI3K-AKT-mTOR, Ras-MAPK and CDK4 in combination with chemotherapy and radiotherapy (Figure 1).

**Figure 1:**
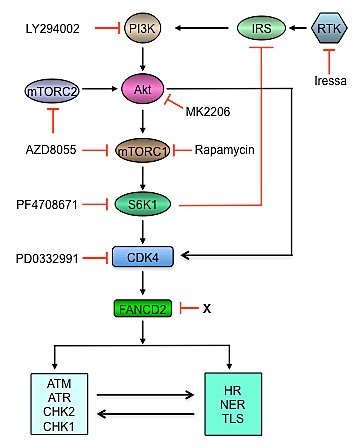
Targeting FANCD2 for sensitization of cancers to radiotherapy and chemotherapy by molecular inhibition of the PI3K-AKT-mTOR signaling pathway In response to DNA damage or replicaiton stress, ATM/CHK2 and ATR/CHK1 checkpoints are activated, thereby promoting DNA damage repair via multiple mechanisms including NER, HR and TLS. DNA damage repair intermediates activate ATM/CHK2 and ATR/CHK1 checkpoints. FANCD2-mediated signaling pathway is one of the key coordinators between ATM/ATR checkpoints and the repair system of NER, HR and TLS. Downregulation of FANCD2 by targeting RTK-PI3K-AKT-mTOR would sensitize cancer cells to DNA damage agents. Directly targeting FANCD2 (marked with X) could be a promising strategy for cancer therapy. AZD8055 (AstraZeneca), mTOR kinase inhibitor; MK2206 (Merck), AKT kinase inhibitor; PD0332991 (Pfizer), CDK4/6 kinase inhibitor; PF4708671 (Pfizer), S6K1 kinase inhibitor. NER, nucleotide excision repair; HR, homologous recombination; TLS, translesion DNA synthesis; RTK, receptor tyrosine kinase; IRS, insulin receptor substrate.

## References

[1] Moldovan GL, D'Andrea AD (2009). Annu. Rev. Genet.

[2] Joo W, Xu G, Persky NS, Smogorzewska A (2011). Science.

[3] Knipscheer P, Raschle M, Smogorzewska A (2009). Science.

[4] Shen C, Oswald D, Phelps D (2013). Cancer Res.

[5] Matsuoka S, Ballif BA, Smogorzewska A (2007). Science.

[6] Liu P, Cheng H, Roberts TM (2009). Nat. Rev. Drug Discov.

[7] Shen C, Lancaster CS, Shi B (2007). Mol. Cell. Biol.

[8] Hoskins EE, Gunawardena RW, Habash KB (2008). Oncogene.

